# The Ribosome as a Switchboard for Bacterial Stress Response

**DOI:** 10.3389/fmicb.2020.619038

**Published:** 2021-01-08

**Authors:** He Cheng-Guang, Claudio Orlando Gualerzi

**Affiliations:** College of Life Sciences, JiLin Agricultural University, Changchun, China

**Keywords:** protein paralogs, translational bias, Cold shock proteins, translation initiation factors, ppGpp

## Abstract

As free-living organisms, bacteria are subject to continuous, numerous and occasionally drastic environmental changes to which they respond with various mechanisms which enable them to adapt to the new conditions so as to survive. Here we describe three situations in which the ribosome and its functions represent the sensor or the target of the stress and play a key role in the subsequent cellular response. The three stress conditions which are described are those ensuing upon: a) zinc starvation; b) nutritional deprivation, and c) temperature downshift.

## Introduction

The ribosome is a gigantic ribozyme entirely devoted to decoding the genetic message and to the synthesis of proteins. These activities ultimately represent the main and most energy demanding functions of bacterial cells whose life mission is to accumulate proteins, grow and proliferate by dividing upon having reached a critical mass. The function of bacterial ribosomes in the initiation, peptide bond formation, elongation and termination steps of translation have been described in review articles, e.g., Rodnina et al. (2007), Korostrelev (2011), and Gualerzi and Pon (2015).

In addition to playing this vital role, ribosomes and, more generally, ribosomal functions are objects and actors in a large number of regulatory circuits. In this article we focus on the role played by ribosomes and the translational machinery in the cellular response to three different types of stress, namely zinc starvation, nutritional deprivation and cold shock. The common denominator of these three stress situations is the central role played by the translational apparatus in the cellular responses, whereas the quite different mechanisms by which it operates offer a wide range of the diverse ways by which ribosomes allow the cell to cope with various environmental cues.

### Participation of the Ribosome in Cellular Zinc Homeostasis

Numerous vital processes such as DNA replication, transcription, translation, electron transfer, and alleviation of oxidative stress, require some transition metals as cofactors. Furthermore, bacterial pathogens must compete with their hosts for limited metal availability resulting from nutritional immunity of the host which restricts metal bioavailability (Kehl-Fie and Skaar, 2010; Cerasi et al., 2013; Becker and Skaar, 2014). On the other hand, because an excess of these metals may have toxic effects, bacteria have evolved a number of mechanisms which ensure metal homeostasis, allowing them to thrive between metal scarcity and toxicity.

Zinc is probably the most important among the essential metal ions (Fe^2+^, Mn^2+^, Cu^2+^, and Ni^2+^), being present in many proteins involved in fundamental processes. In fact, zinc is a structural or catalytic cofactor of many enzymes, including the metallo-beta-lactamases which inactivate beta-lactam antibiotics playing an important role in infections caused by multi-drug resistant bacteria (Kim et al., 2011, 2013). One of the enzymes whose activity requires zinc is GTP cyclohydrolase I (GCYH-I), the first enzyme in the pathway of tetrahydrofolate (THF) biosynthesis. Thus, as detailed below, zinc availability can affect THF levels thereby impacting on the translational activity of the cell.

Zn^2+^ is used because of its peculiar and favorable chemical properties and during catalysis behaves like a Lewis acid, accepting electron pairs or attracting/stabilizing negative charges of substrates (Andreini et al., 2008).

Approximately 6% of *Escherichia coli* proteins are able to bind zinc (Andreini et al., 2008) and proteomic studies have shown that several ribosomal proteins (i.e., L2, L13, S2, S15, S16, and S17) can bind Zn (Katayama et al., 2002). Furthermore, another biophysical study (Hensley et al., 2012) showed that in *E. coli* eight Zn equivalents are bound to ribosomal proteins S2, S15, S16, S17, L2, L13 and to the highly conserved 50S proteins L31 and L36 (Wada, 1986a,b; Wada and Sako, 1987) which contain a Zn ribbon motif consisting of four cysteines (histidines in some cases) (Makarova et al., 2001; Panina et al., 2003). As detailed below, these proteins play an important role upon Zn starvation, acting as cellular Zn^2+^ reservoirs (Akanuma et al., 2006).

Protein L31 bridges the 30S head and central protuberance of the 50S subunit and, by virtue of its dynamic nature, maintains its interactions with both subunits during ribosome ratcheting movement as its linker region switches from an extended to a kinked conformation (Fischer et al., 2015). During stationary phase, under the influence of the ribosome modulation factor, L31 also plays a role in 100S (dimers of 70S ribosomes) formation (Wada et al., 1990). In ribosomes lacking L31, peptide elongation is slower, subunit association weakened and no 100S are formed. Furthermore, although L31 is not essential for survival, strains lacking this protein grow very slowly and their ribosomes are 40% less active than those having one copy of L31 (Ueta et al., 2017).

Protein L36, the smallest, most basic and widely conserved ribosomal protein belongs to the late (“fifth”) assembly group identified by mass spectrometry (Chen and Williamson, 2013), being involved in the late steps of 50S subunit assembly upon binding to 23S rRNA (Urlaub et al., 1995). Together with the RlmE-dependent U2552 2′-*O*-methylation, L36 promotes the association between helices 92 and 71 resulting in the structural organization of 23S rRNA (Arai et al., 2015). Cells lacking L36 are viable but between 30 and 42°C display a 50% growth retardation compared to wt cells (Maeder and Draper, 2005). Despite displaying normal 30S–50S association and 100S formation during stationary phase, ribosomes lacking L36 have a 30% reduced translational activity.

**FIGURE 1:**
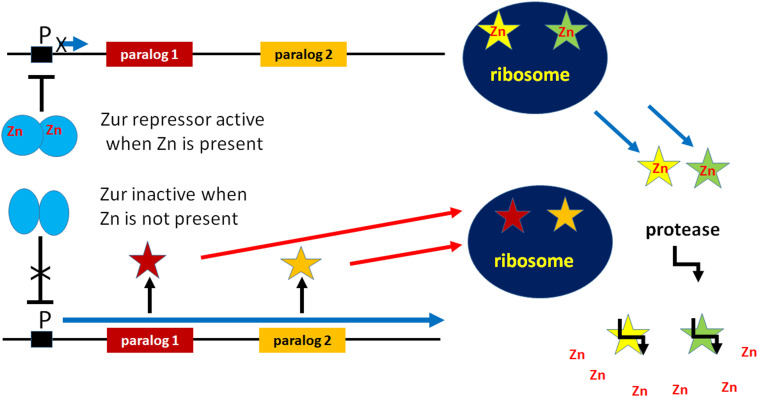
Participation in cellular Zn homeostasis of non-Zn-containing paralogs of ribosomal proteins. Under conditions of Zn deprivation, the Zn-dependent transcriptional repressor Zur is inactivated allowing the expression of non-Zn-containing paralogs of Zn-containing ribosomal proteins. These paralogs bind to the ribosome and functionally replace the Zn-containing proteins which are freed and degraded by proteolysis so as to free Zn into the cytoplasm.

Interestingly, in *E. coli* there are two proteins, YkgM and YkgO, which are paralogs of L31 and L36, respectively. YkgM (87 residues) is 17 residues longer than L31 while YkgO is eight amino acids longer than L36 (46 vs 38 residues). Unlike L31 and L36, these paralogs do not contain a Zn-binding motif. Paralogs of L31 and L36 lacking zinc-binding sites have been detected in many gamma-proteobacteria and in *Streptomyces*. In *E. coli* L31 and its paralog YkgM are encoded by *rpmE* and *ykgM*, respectively, while L36 and its paralog YkgO are encoded by *rpmJ* and *ykgO*, respectively. As illustrated in Figure 1, these genes are Zn-regulated insofar as only *rpmE* and *rpmJ* encoding L31 and L36 are expressed in the presence of zinc, whereas *ykgM* and *ykgO*, which encode the paralogs, are expressed only when zinc is limiting. *YkgM* and *ykgO* are present in the same operon whose transcription is inhibited by the Zur repressor, a member of the Fur family of metal-responsive regulators. In the presence of zinc a dimer of Zur dimers recognizes and binds a 13 bp palindrome with a three-base spacer (RNNNYxxxRNNNY) (Gilston et al., 2014). However, Zur becomes inactivated upon zinc depletion and zinc uptake genes of the ABC family of transporters such as AdcABC of Gram positive and ZnuABC of Gram-negative bacteria are derepressed (Panina et al., 2003; Hantke, 2005; Graham et al., 2009; Hemm et al., 2010; Velasco et al., 2018). Likewise, transcription of the *ykgM*-*ykgO* operon is induced and YkgM and YkgO are expressed (Graham et al., 2009; Hemm et al., 2010) and replace their paralogs L31 and L36 upon binding to the same ribosomal site (Ueta et al., 2020). Ribosomes containing one copy of YkgM and no L31 are functionally equivalent to wild-type ribosomes which contain one copy of L31 as far as subunits association, *in vitro* translation and 100S formation are concerned. Furthermore, mutants carrying these ribosomes grow like wt cells indicating that YkgM is functionally identical to L31 and that the zinc bound to L31 is not involved in translational processes.

Protein L36 contacts the 23S rRNA at sites which are relevant for the peptidyl transferase center (H89 and H91) and for elongation factor binding (H42, the stem that binds L10/L12 and H95 in the four-helix junction containing the sarcin-ricin loop). The tertiary interactions between helices 89, 91, and 95 are disrupted in ribosomes lacking L36. In spite of this, *rpmJ* is not essential for growth in L-broth at 37°C. However, whereas cells expressing the paralog YkgO display wild-type growth, cells lacking *rpmJ* grow more slowly indicating that L36 can be functionally replaced by its paralog YkgO, at least as far as growth is concerned (Maeder and Draper, 2005; Baba et al., 2006).

All evidence gathered so far indicates that the presence of zinc in the two ribosomal proteins is not necessary for the translational function of the ribosome because L31 and L36 can be replaced by the functionally equivalent non-Zn-containing paralogs YkgM and YkgO. Thus, it can be surmised that L31 and L36 are related to Zn storage and provision. In fact, under conditions of zinc starvation, the non-Zn containing paralogs of L31 and L36 are expressed and incorporated into ribosomes in place of the original Zn-containing ribosomal proteins. Under conditions of Zn shortage, instead of using all available zinc, the proteins containing the Zn ribbon act as Zn suppliers, being excluded from the ribosomes and degraded so that Zn is released into the cytoplasm for utilization by other vitally important zinc-binding proteins such as DNA polymerase, primase, etc. (Panina et al., 2003; Nanamiya et al., 2004; Gabriel and Helmann, 2009) (Figure 1).

As to the efficacy of this system, it should be considered that the number of ribosomes, each containing one copy of L31 and L36, is thousands fold higher than that of other cellular Zn-requiring proteins and that the Zn concentration needed by the cell is very low. Indeed, the concentration of readily exchangeable zinc measured in *E. coli* cells was found to vary significantly depending upon the extracellular zinc concentration, but to be in the pmolar range (i.e., in average ca. 2 × 10^–11^ M) (Outten and O’Halloran, 2001; Wang et al., 2011).

A situation similar to that just described for *E. coli* is likely common to other bacterial species, although other Zn-binding ribosomal proteins, such as L33 and S14, might be involved. Indeed, several bacterial genomes encode paralogs of ribosomal proteins L36, L33, L31, and S14 which lack the Zn-ribbon and are expressed upon zinc starvation. When L31-paralogs are not present, like in some Staphylococci, Streptococci, Listeriae etc. the genome encodes L33 and S14 paralogs. Remarkably, the *Bacillus subtilis* genome encodes paralogs of all these proteins (i.e., L36, L33, L31, and S14) (Panina et al., 2003).

In conclusion, it appears clear that bacterial cells respond to zinc shortage with inactivation of the Zn-dependent transcriptional repressor Zur, resulting in the expression not only of genes encoding high-affinity Zn transporters, such as ZnuABC, but also of the non-Zn containing paralogs of ribosomal proteins L31, L33, L36, and S14 which participate in maintaining cellular Zn homeostasis (Hantke, 2005; Moore and Helmann, 2005).

**FIGURE 2:**
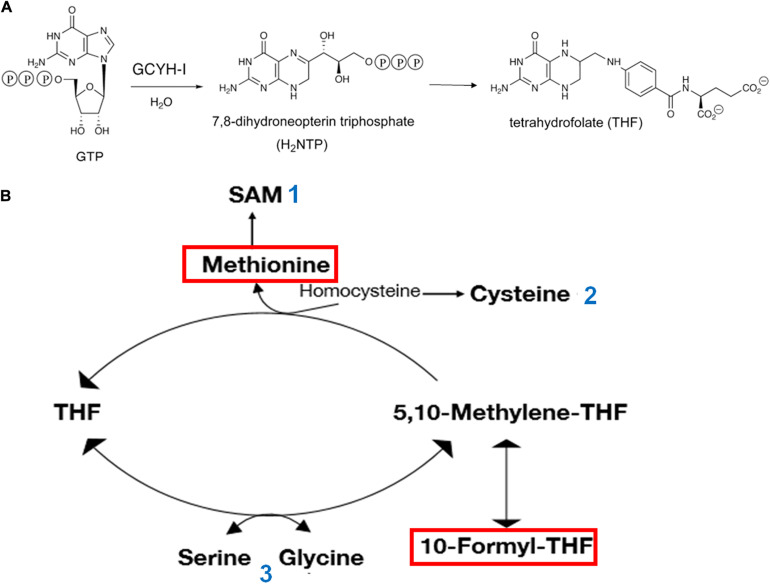
Involvement of Zn and GTP in THF synthesis and central role of 1-C metabolism in translation initiation. **(A)** The Zn-dependent enzyme GTP cyclohydrolase I (GCYH-I) catalyzes the conversion of GTP to 7,8-dihydroneopterin triphosphate which in turn is converted to 6-hydroxymethyl-dihydropterin by dihydroneopterin aldolase (FolB) and finally to THF through the consecutive action of enzymes FolK, FolP, FolC, and FolA. **(B)** The amino acid methionine, used for the aminoacylation of methionine-specific elongator and initiator tRNAs and 10-formyl-THFMethyl, which transfers a formyl group to the α-NH_2_ group of methionine in Met-tRNA_*fMet*_ are boxed in red. Additional molecules playing a role in the translational apparatus are shown. The numbers next to these molecules indicate their participation as: 1 – methyl donor for all types of RNA molecules and proteins; ribosyl donor for queuosine modification of the first anticodon position of tRNAs specific for His, Asp, Asn, and Tyr; synthesis of polyamine; 2 – aminoacylation of tRNA specific for Cys; thio-modification of tRNAs; 3 – aminoacylation of tRNAs specific for Ser and Gly.

As mentioned above, variations of Zn availability can affect one-carbon (1-C) metabolism and, in turn, translational activity of the cell. In fact, the first enzyme of the pathway leading to THF biosynthesis is the Zn-dependent GTP cyclohydrolase I (GCYH-I). In addition to being involved in transferring 1-C units to purines, thymidylate, pantothenate, glycine and serine, THF is involved in the synthesis of methionine and formylmethionyl-tRNA which are essential elements of the translation initiation pathway. The Zn-dependent GCYH-I catalyzes the conversion of GTP to 7,8-dihydroneopterin triphosphate (Figure 2A) in a complex reaction that begins with hydrolytic opening of the purine ring at C-8 of GTP to generate an N-formyl intermediate. The role of Zn^2+^ in the first reaction step consists in the activation of a water molecule for nucleophilic attack at C-8 (Green et al., 1996). While GCYH-I is encoded by *folE* in *E. coli*, another type of GCYH-I enzyme, named GCYH-IB, has been detected in approximately 25% of the bacterial species whose genomes have been sequenced. This enzyme, encoded by *folE2*, does not require Zn but can be activated by a variety of other divalent cations (Sankaran et al., 2009). Although the majority (i.e., ca. 2/3) of bacteria possess either a *folE* (like *E. coli*) or *folE2*, a sizable minority of bacteria, *B. subtilis* among them, possesses both genes; it is interesting that whereas *folE* is expressed in the presence of sufficient levels of Zn, *folE2* is repressed by the Zn^2+^-dependent Zur repressor and is expressed under Zn-limiting conditions allowing folate biosynthesis even during Zn starvation. This underscores both importance of systems ensuring zinc homeostasis and the vital role played by THF in the cells (Figure 2B).

### Central Role of Ribosomes in the Response to Nutritional Stress

Translation initiation is generally regarded as being rate limiting for bacterial protein synthesis and therefore represents the step which is preferentially and more frequently subjected to post transcriptional regulation (McCarthy and Gualerzi, 1990).

In addition to the energetic costs connected with synthesis and assembly of the translational apparatus, protein synthesis itself uses a considerable share of the cellular energy pool as large amounts of ATP and GTP are required to activate amino acids for tRNA aminoacylation and to support the activity of the translational GTPases (IF2, EF-Tu, EF-G, and RF3). To prevent superfluous use of energy when nutrients are in scarce supply, translation must be tightly regulated at the initiation step which depends upon the metabolic state of the cell, upon 1-C metabolism and upon the nature and concentration of guanine nucleotides.

**FIGURE 3:**
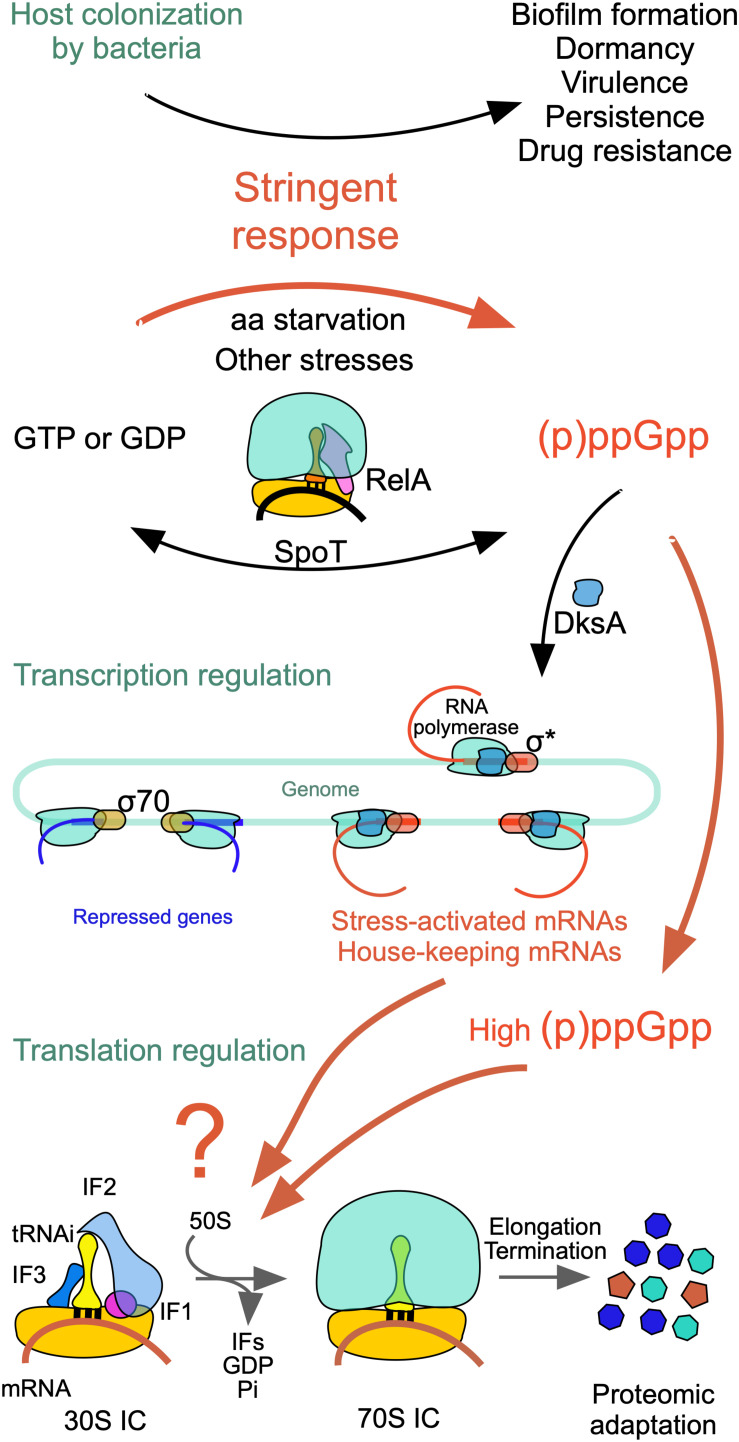
Scheme of the events triggered by nutritional stress. The stringent response modifies cell physiology so as to affect biofilm formation, dormancy, virulence, persistence and drug resistance. Ribosome-associated proteins RelA and SpoT synthesize large amounts of (p)ppGpp and ppGpp which affect negatively σ^70^-dependent transcription of genes subjected to stringent control while allowing the transcription of σ^*S*^-dependent housekeeping genes. In addition, ppGpp or (p)ppGpp bind to IF2 in place of GTP resulting in the selective inhibition or stimulation of mRNA translation with a consequent modification of the cellular proteome. For additional information see text and Vinogradova et al. (2020) from which this figure is taken.

Under optimal growth conditions, GTP is present in millimolar concentrations in the cell, whereas the very low levels of the alarmones (p)ppGpp (guanosine 3′-diphosphate,5′-triphosphate) and ppGpp (guanosine 3′,5′-bispyrophosphate) play essential roles in various aspects of bacterial physiology, modulating growth rate, general metabolism and GTP homeostasis (Potrykus and Cashel, 2008; Potrykus et al., 2011; Kriel et al., 2012; Hauryliuk et al., 2015; Fernández-Coll and Cashel, 2020). However, under a variety of stress conditions, including starvation for an essential amino acid, which gives rise to a “stringent response,” many bacteria synthesize large amounts of these alarmones (Figure 3). During stringent response, the ppGpp concentration increases ca. 100-fold reaching 1 mM in *E. coli* but substantially less in *B. subtilis* (Kriel et al., 2012). The accumulation of (p)ppGpp has drastic effects on various vital functions of the cell (Figure 3) and in some cases, like in *B. subtilis* (but not in *E. coli*), is accompanied by a substantial reduction of the cellular level of GTP. In turn, a reduction of GTP levels affects the 1-C metabolism, THF synthesis and ultimately translation (Figure 2).

**FIGURE 4:**
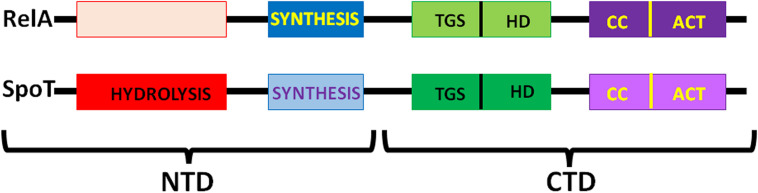
Schematic representation of the six-domain structure of *E. coli* RelA and SpoT. From left (NTD) to right (CTD) the proteins contain: the (p)ppGpp hydrolysis domain active in SpoT and inactive in RelA; the (p)ppGpp synthesis (SYNTH) domain, more active in RelA than in SpoT; the ThrRS, GTPase, and SpoT (TGS) domain; the helical domain (HD); the conserved cysteines domain (CC) and the Aspartokinase, Chorismate mutase and TyrA domain (ACT). Additional details can be found in the text and in Atkinson et al. (2011).

Synthesis of (p)ppGpp and ppGpp entails the transfer of the ATP pyrophosphate to either GDP or GTP (Chatterji and Ojha, 2001) and occurs through the action of RelA/SpoT homologue (RSH) proteins which are highly conserved in bacteria and consist either of a single domain or of multiple domains as in *E. coli*. RelA and SpoT have a similar architecture (Figure 4); the catalytic module responsible for (p)ppGpp synthesis is located in the *N*-terminal (NTD) region and is more active in RelA than in SpoT whereas the module responsible for (p)ppGpp hydrolysis is active in SpoT and inactive in RelA (Atkinson et al., 2011). The CTD region of RelA has regulatory functions, as detailed below. Protein RelA associates with the 50S subunit and its function is partially dependent upon ribosomal protein L11 (Ramagopal and Davis, 1974; Gropp et al., 2001; Yang and Ishiguro, 2001; Wendrich et al., 2002). The CTD is buried inside the ribosome while the catalytic region is exposed to the cytoplasm. Sensing the binding of an uncharged cognate tRNA to the A-site of stalled ribosomes promotes the stabilization of the TGS domain (Figure 4) in a position in which the uncharged CCA 3′ end of tRNA can fit in a pocket. Synthesis of (p)ppGpp is triggered when the contact between the NTD and ribosome is stabilized by a contact of the synthetase domain with the tip of the 16S rRNA spur, while the non-functional hydrolase domain is bound near the 23S rRNA sarcin-ricin loop (Arenz et al., 2016; Brown et al., 2016; Loveland et al., 2016; Winther et al., 2018).

Unlike RelA, SpoT is a bifunctional protein responsible for both synthesis and hydrolysis of (p)ppGpp. Synthesis of (p)ppGpp by SpoT is triggered by a variety of cellular stress conditions (Figure 3) whereas ribosomes and uncharged tRNA inhibit its hydrolytic activity (Richter, 1980). In spite of some past controversy concerning the SpoT location, more recent data have shown that SpoT interacts with the ribosome-associated GTPase CgtA and is also associated with a pre-50S particle independent from CgtA. In fact, CgtA promotes (p)ppGpp hydrolysis by SpoT on the ribosome whereas its loss from the ribosome is necessary for maximal (p)ppGpp accumulation under stress conditions (Jiang et al., 2007).

The increase in the ppGpp concentration has pleiotropic effects, modulating expression, stability, oligomerization and activity of a variety of proteins and regulatory RNAs, inhibiting DNA synthesis and causing cell cycle arrest (Magnusson et al., 2005; Maciąg-Dorszyńska et al., 2013 and references therein) (Figure 3). Effects of ppGpp and pppGpp on transcription are also well documented (Potrykus and Cashel, 2008; Potrykus et al., 2011). Both alarmones bind in a small, positively charged cavity on the outer surface of RNA polymerase, at the interface between the β′ (which binds the guanosine) and ω subunits (which binds both pyrophosphates). Because the binding site is located far away (i.e., 30 Å) from the polymerization center, regulation of the RNAP activity by ppGpp is believed to depend upon an allosteric mechanism (Mechold et al., 2013; Zuo et al., 2013) thereby regulating promoter selection (Chatterji et al., 1998; Toulokhonov et al., 2001; Magnusson et al., 2005; Paul et al., 2005). This binding results in the selective transcriptional inhibition of genes subjected to stringent control such as those encoding rRNA and tRNA as well as genes required for growth and proliferation while allowing expression of genes which encode products enabling physiological adaptations and ultimately cell survival, such as amino acid biosynthesis genes (Potrykus and Cashel, 2008) (Figure 3). The basis for the selective silencing of some genes, while other genes are expressed, can be found at the level of both transcription and translation, as will be detailed below.

In addition to their direct participation in the stringent response as carriers of RelA and SpoT, which are responsible for ppGpp accumulation, the ribosomes play additional roles in the cellular response to nutritional stress. In fact, a shortage of carbon, nitrogen and sulfur can drastically affect ribosomal functions during translation initiation.

Indeed, the use of methionine as the *N*-terminal amino acid of nascent polypeptides and the modification of its α-NH_2_ group with a formyl group derived from 10-formyl-tetrahydrofolate tightly connect translation initiation to folic acid-mediated 1-C metabolism whose main outputs are methionine and AdoMet. In turn, methionine is a major donor of 1-C units in the cell and plays an essential role in a large number of methylations. Furthermore, together with cysteine, methionine is the only sulfur containing amino acid.

As mentioned above, protein synthesis initiates with a methionine residue esterified to the 3′ acceptor end of a special tRNA molecule (i.e., tRNA_*fmet*_). The peculiar characteristics of this tRNA are recognized by a specific transformylase which transfers a formyl group from 10-formyl-tetrahydrofolate to the α-NH_2_ group of methionine to yield initiator fMet-tRNA_*fMet*_ (Mangroo et al., 1995; RajBhandary and Chow, 1995; Mechulam et al., 1999; Mayer et al., 2001). In turn, fMet-tRNA_*fMet*_ is used to initiate protein synthesis in the majority of the cases. As a result, formyl-methionine is almost invariably the *N*-terminal amino acid of bacterial nascent peptides. A possible exception could be the case of *Firmicutes* which may initiate protein synthesis in the absence of folate-mediated formylation of methionyl-tRNA_*fMet*_ when grown in a medium supplemented with all the metabolites directly associated with 1-C metabolism (e.g., serine, glycine, purines, thymine, and pantothenate) (Samuel et al., 1970).

**FIGURE 5:**
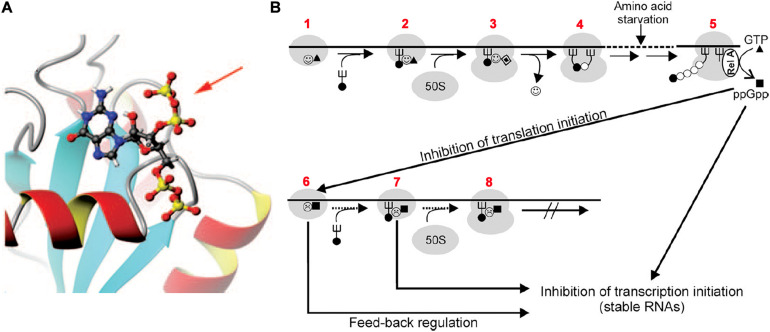
Translation initiation inhibition by ppGpp binding to initiation factor IF2. **(A)** 3D NMR structure of the G-2 domain of *Geobacillus stearothermophilus* IF2 complexed with a ppGpp molecule (represented by “balls and sticks”). The 3′ diphosphate protruding out of the IF2 domain is indicated by a red arrow. **(B)** Scheme of the postulated regulatory circuits involving IF2 and ppGpp upon nutrient starvation. Steps: 1–2 – formation of 30SIC in the presence of IF2-GTP (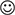
▲), 2–3 – association of 50S subunit and formation of 70SIC and GTP (▲) hydrolysis to GDP (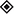), 3–4 – IF2 (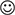) dissociation and initiation dipeptide formation. 5 – Amino acid starvation during elongation triggers RelA-dependent synthesis of ppGpp (■), which inhibits the translation initiation functions of IF2 (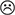) such as 30SIC and initiation dipeptide formation, generating also a feed-back inhibition of stable RNA transcription since IF2, fMet-tRNA, and ppGpp were found to interact with the RNA polymerase and influence its activity at stable RNA promoters (Debenham et al., 1980; Travers et al., 1980), while IF2 (Cole et al., 1987), IF3 (Olsson et al., 1996), and initiation-competent 30S subunits (Yamagishi et al., 1987) were shown to be required for feedback repression of stable RNA transcription. Figures taken from Milon et al. (2006). Copyright (2006) National Academy of Sciences.

FMet-tRNA_*fMet*_ is recognized and bound by the *C*-terminal domain of IF2, the largest of the three initiation factors (Guenneugues et al., 2000; Spurio et al., 2000), and recruited to the ribosomal P site by the ribosome-bound factor (Milon et al., 2010). IF2 is a G protein whose G2 domain (Wienk et al., 2012) binds and hydrolyzes GTP in the presence of aliphatic alcohols or upon activation by its interaction with the ribosomal GTPase-associated center consisting of the 23S rRNA sarcin-ricin loop and ribosomal protein L7/L12 (Gualerzi et al., 1991; Severini et al., 1991; La Teana et al., 2001; Diaconu et al., 2005; Koch et al., 2015; Carlson et al., 2017). In addition to GTP, also GDP and the alarmone ppGpp can bind to the same site of IF2 (Figure 5A) with similar or somewhat greater affinities. However, unlike GTP, which strengthens the IF2 interaction with the 30S subunit and stimulates the functions of the factor, GDP interferes with these activities whereas ppGpp severely inhibits them (Pon et al., 1985; Fabbretti et al., 2012) (Figure 5B). In fact, it has been shown that ppGpp inhibits translation by blocking the IF2-dependent formation of the 30S initiation complex (30SIC). Although ppGpp can also bind to elongation factors EF-G and EF-Tu, translation inhibition was shown to be selectively caused by inhibition of the initiation factor (Milon et al., 2006). This conclusion is in full agreement with the finding that ppGpp has hardly any effect on the activity of EF-Tu (Pingoud et al., 1983; Rojas et al., 1984) and that ppGpp displays a ∼5-fold higher affinity for IF2 than for EF-G suggesting that “during stringent response *in vivo*, IF2 is more responsive to ppGpp than to EF-G” (Mitkevich et al., 2010).

Based on these findings it was suggested that a fundamental *raison d’être* of the guanosine nucleotide binding site of IF2 is to confer upon this factor the properties of a metabolic sensor capable of causing a feedback inhibition by blocking *de novo* protein synthesis when the nutritional conditions become unfavorable (Milon et al., 2006) (Figure 5B).

Selective inhibition by ppGpp of the initiation functions of IF2, such as 30SIC formation and docking of the 50S subunit to yield a 70SIC, was confirmed in a recent study in which it was shown that inhibition of IF2 activity by ppGpp occurs also during cellular entry into quiescence (Diez et al., 2020).

As mentioned above, the stringent response entails not only the silencing of some genes whose expression would be detrimental under nutritional stress, but also the activation of other genes which allow cells to cope with the unfavorable situation. This implies that both transcription and translation are not indiscriminately inhibited (Figure 3). Accordingly, a recent study, while confirming the previous finding that ppGpp-bound IF2 inhibits translation by shifting to the left the 30S pre-IC ⇆ 30SIC equilibrium (Milon et al., 2006), showed that ppGpp does not inhibit the translation of all mRNAs but instead allows the synthesis of a select group of proteins (Vinogradova et al., 2020). This is possible because the affinity of GTP for IF2 was shown to depend upon the type of mRNA present in the 30S pre-IC. Thus, when the affinity of the factor for GTP is very high, ppGpp cannot compete for the 30S-bound IF2 and translation can occur. In this study it was shown that *tufA* mRNA, expressing elongation factor EF-Tu, enhances the affinity of GTP for 30S complexes thereby increasing the tolerance for ppGpp and allowing efficient translation. On the contrary, the GTP affinity in complexes containing *infA* mRNA, expressing initiation factor IF1, was low enough to allow ppGpp to compete with GTP and to block translation initiation (Vinogradova et al., 2020) (Figure 3). The peculiar feature responsible for enhancing GTP affinity and conferring ppGpp tolerance was shown to be the presence of a structured enhancer of translation initiation (SETI) consisting of two consecutive hairpins proximal to the translation initiation region (TIR) of *tufA* mRNA as well as of several other enterobacterial mRNAs. Furthermore, it was suggested that, whereas the primary sequence of the SETIs is irrelevant, an important role is played by the spatial or conformational constraints which they impose on the initiation complex. Finally, another interesting finding of this study is that IF2 can use the alarmone pppGpp to promote translation initiation, albeit with a reduced efficiency (Figure 3).

### Ribosomal Functions Drive the Global Response to Cold Stress

**FIGURE 6:**
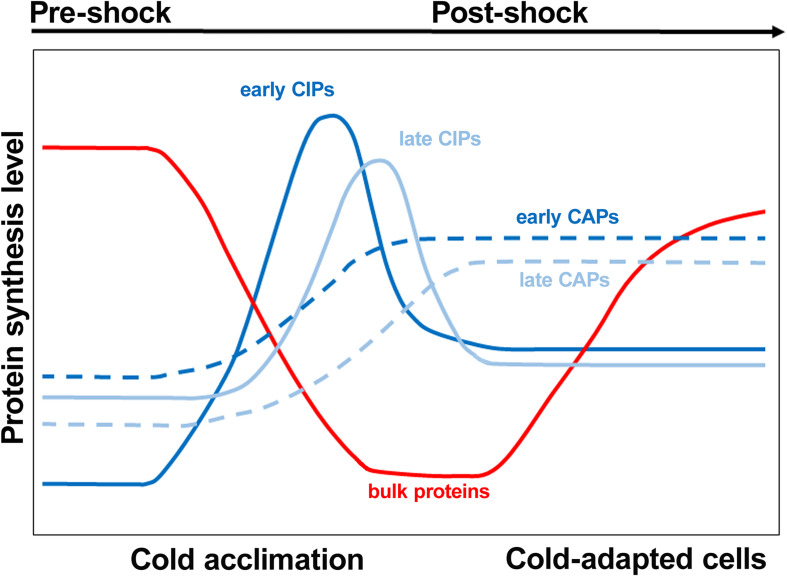
Cold shock induced proteins are expressed at different times after the stress. During the initial stage immediately following cold exposure, the rate of bulk protein synthesis drops while early cold shock proteins such as CspA are synthesized. Subsequently, a group of late cold shock proteins such as polynucleotide phosphorylase starts to accumulate. As the cold acclimation phase nears the end, the levels of cold shock proteins decline while early and late proteins typical of cold-adapted cells are synthesized. Bulk protein synthesis and cell growth resume at the end of the acclimation period. Modified from Weber and Marahiel (2003). Copyright (2003) SAGE publishing.

An abrupt lowering of the temperature below the optimal growth condition is one of the most frequent stressful occurrences experienced by bacterial cells. Regardless of whether the bacteria are thermophilic (Wu and Welker, 1991), mesophilic (Jones et al., 1987; Lottering and Streips, 1995; Phan-Thanh and Gormon, 1995) or psychrophilic (Cloutier et al., 1992; Whyte and Inniss, 2011), a drastic lowering of the ambient temperature causes a “cold shock,” which entails extensive reprogramming of the gene expression pattern. Cellular response to cold stress has been extensively studied in the two paradigm Gram negative and Gram positive bacteria, *E. coli* (Jones and Inouye, 1994; Gualerzi et al., 2003, 2011; Giuliodori, 2016) and *B. subtilis* (Graumann et al., 1996, 1997), respectively. In the mesophile *E. coli*, lowering the temperature from 37°C to <20°C causes an immediate block of all macromolecular syntheses, except for a few gene products which are essential for cold adaptation and eventually cell survival (Broeze et al., 1978; Jones et al., 1987; Gualerzi et al., 2003). The precise number of proteins whose expression is increased following cold stress remains somewhat elusive. A set of at least 26 cold-shock genes preferentially and transiently expressed after the stress has been listed for *E. coli* (Gualerzi et al., 2003), 37 for *B. subtilis* (Graumann et al., 1996), and 32 for *Streptomyces aureofaciens* (Mikulik et al., 1999). More recently, a thorough ribosome profiling study identified 116 *E. coli* genes that are upregulated ≥2-fold after cold stress (Zhang et al., 2018). In any event, as seen in the scheme presented in Figure 6, not all cold shock proteins are expressed at the same time; there are cold-induced proteins such as CspA and the three translation initiation factors, which are expressed at an early stage following the stress while others, such as polynucleotide phosphorylase, which are expressed at a later stage. In addition, there are proteins which selectively accumulate in cold-adapted cells. The nature of the cellular receptors responsible for sensing temperature downshift is not clear, but because temperature changes affect a large number of physical and chemical parameters in the cell, it is likely that there might be multiple ways in which thermal stress can be sensed by the cell. In particular, structural modifications of nucleic acids and proteins induced by temperature changes are likely involved in triggering a cascade of events which allow bacteria to adapt to low temperature. Two typical cases in which low temperature-induced changes of nucleic acid structures result in changes of the gene expression pattern are the *virF* promoter of *Shigella flexneri* and the transcript of the *cspA* gene of *E. coli*. In the case of *virF*, the gene which controls the pathogenicity cascade of *S. flexneri*, the promoter contains a curved stretch of DNA which represents the target of the transcriptional repressor H-NS. However, the curvature disappears above a threshold temperature so that H-NS fails to bind and to block transcription (Falconi et al., 1998). In the case of *cspA*, the mRNA responds to temperature changes by adopting different functional structures. The mRNA in the cold-shock structure is translated more efficiently and is less prone to degradation than the *cspA* mRNA in the 37°C structure. As a result, immediately after cold stress large amounts of CspA protein are synthesized (Giuliodori et al., 2010).

Most of the regulatory mechanisms involved in the cellular response to cold shock occur at the post-transcriptional level; among these mechanisms the increased half-lives of the transcripts of cold shock genes (Goldenberg et al., 1996) and modifications of the translational apparatus appear to be particularly relevant (Gualerzi et al., 2003). Indeed, there are data suggesting that the ribosome itself might act as a physiological sensor for the thermal stress. In fact, it has been shown that synthesis of stress-related proteins can be induced by the administration of antibiotics which target the ribosome such as chloramphenicol (cold-shock proteins) and kanamycin (heat-shock proteins) (Van Bogelen and Neidhardt, 1990). Aside from this, the ribosomes and the translational apparatus play a fundamental role in the cellular response to cold stress and in the selective translation of cold shock mRNAs while bulk protein synthesis is shut down. In this connection, it was shown that after cold shock, even if present in large amounts in the cell, transcripts of non-cold shock genes are not translated, unlike transcripts of cold shock genes (Brandi et al., 1996).

The selective expression of cold shock proteins while translation of non-cold shock mRNAs is inhibited was termed “translational bias” and its molecular basis was traced back to a modification of the translational apparatus. In particular, translational bias was shown to be due to both *cis*-acting and *trans*-acting elements.

Among the *cis*-acting elements there are the sequences of *cspA* mRNA, the main cold shock transcript whose structure, as mentioned above, changes at the cold shock temperature acquiring a conformation which makes it better suited for translation in the cold and more resistant to degradation (Giuliodori et al., 2010). The elements present in the cold-shock transcripts which render these mRNAs suitable for translation in the cold were identified analyzing the translational activity of chimeric mRNAs constructed by shuffling regions of *cspA* and *cspD* mRNAs representing the paradigm cold shock and non-cold shock transcripts, respectively. From these studies it was concluded that, at least in the case of *cspA* mRNA, the cold-responsive elements are located within the first 50 bases of the 5′UTR and in the region immediately downstream the initiation triplet (Giuliodori et al., 2019).

**FIGURE 7:**
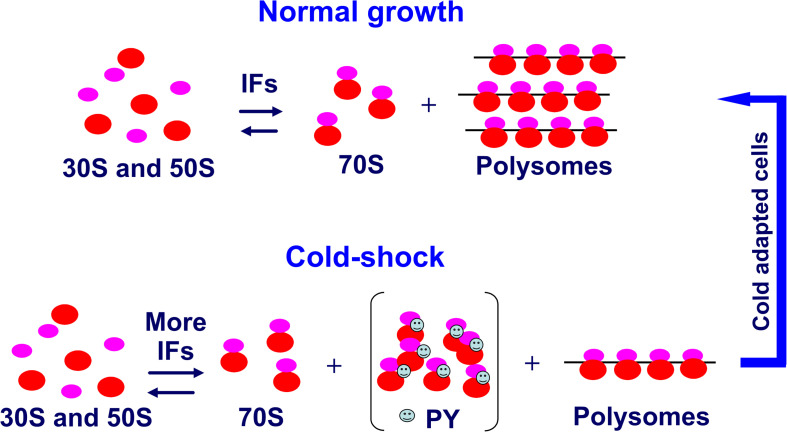
Role of increased IF levels in maintaining a pool of translation initiation competent ribosomal subunits. Compared to the pre-stress condition, after cold stress the cell requires a reduced number of translating ribosomes (polysomes). The cold-shock induced protein PY or in its absence perhaps the ribosome modulating factor (Wada et al., 1990) sequesters a large proportion of the 70S monomers derived from polysome dissociation and stabilizes them in a functionally idle state. The few potentially active 70S monomers would tend to remain stably associated at low temperature unless an increased level of IF3 and IF1 counteracts the increased tendency of the ribosomal subunits to remain associated at low temperature so as to maintain a sufficiently high level of subunits amenable to initiate translation. Taken from Gualerzi et al. (2011).

Concerning the *trans*-acting elements, it was shown that upon temperature downshift, transcription and synthesis of the initiation factors IF1 (Giangrossi et al., 2007), IF2 (Brandi et al., 2019a), and IF3 (Giuliodori et al., 2007) are stimulated, whereas maturation of the rRNA and the assembly of ribosomes slow down considerably (Piersimoni et al., 2016). This results in an imbalance of the initiation factor/ribosome ratio which is indeed of great importance for the events leading to cold acclimation of the cell. Whereas the increased level of IF2 does not contribute to translational bias but is important for its participation in the assembly and maturation of ribosomes during cold adaptation (Brandi et al., 2019b), the increased IF3/70S and IF1/70S ratios are at least in part responsible for translational bias. These factors counter the increased tendency of the ribosomal subunits to associate at low temperature, thereby ensuring a sufficient pool of 30S subunits for promoting 30S initiation complex (30SIC) formation, 50S subunits docking to yield 70SIC and ultimately translation of a select group of cold-shock mRNAs (Giangrossi et al., 2007; Giuliodori et al., 2007) (Figure 7). In particular, IF3 was shown to be the main factor responsible for promoting cold shock mRNA translation and inhibiting non-cold shock mRNAs by targeting the early steps of protein synthesis. This activity of IF3 is strengthened by IF1 (Giuliodori et al., 2004).

Another *trans*-acting factor which affects translation during cold acclimation is CspA. This protein (70 residues in *E. coli*), which contains two RNA-binding motifs, RNP1 (K16GFGFI21) and RNP2 (V30FVHF34) on two different β-strands, binds cooperatively to both single-stranded DNA (ssDNA) and RNA (Jiang et al., 1997). CspA is expressed to very high levels immediately after cold shock so that it was designated as the “major cold shock protein” (Goldstein et al., 1990). However, meticulous studies have later demonstrated that this protein is also present in massive amounts under non-stress conditions, being one of the most abundant proteins during the first rounds of cell division at 37°C. Subsequently, CspA almost disappears from the cells, being diluted in the cytoplasm of the proliferating cells while new synthesis is limited by the extreme instability acquired by its mRNA (Brandi et al., 1999; Brandi and Pon, 2012). The presence of large amounts of CspA in both cold-stressed cells and in cells growing at 37°C indicates that the function of this protein is not restricted to its roles during cold acclimation. Indeed, by virtue of its property of being a single-stranded nucleic acid binding protein, CspA is involved in a number of functions.

Initially, CspA was identified as a factor capable of stimulating transcription of *hns* (La Teana et al., 1991) and *gyrA* (Jones et al., 1992) after cold shock, most likely by keeping single-stranded the DNA in the promoter region, thereby favoring promoter clearance (Brandi et al., 1994). Subsequently, it was shown that purified CspA stimulates also translation *in vitro* (Brandi et al., 1996; Giuliodori et al., 2004), likely by preventing the formation of stable secondary structures in mRNA by virtue of its RNA chaperone activity (Jiang et al., 1997).

It should be mentioned that in addition to CspA, *E. coli* contains eight CspA paralogs, five of which (CspA, CspB, CspG, CspE, and CspI) are cold-shock inducible (Yamanaka, 1999). These proteins have very similar structural and functional properties so that they may have overlapping functions, but whether or not also these proteins are involved in translation during cold adaptation remains an open question.

Finally, the possibility that cold stress may result in some compositional heterogeneity in the ribosomal population was taken into consideration. However, ribosomes extracted from control and cold-shocked cells proved to perform equally well, at least in *in vitro* translational tests, despite the presence of several post-translational modifications in some ribosomal proteins (Piersimoni, 2010).

Another essential aspect of translational bias is the inhibition of the translation of non-cold-shock mRNAs. It has been calculated that 30 min after cold shock the bulk protein synthesis rate in *E. coli* drops ∼200-fold as a result of a 50-fold reduction of the translation elongation rate and of a four-fold decrease of the translation initiation rate (Zhang et al., 2018).

The unfavorable mRNA structures acquired by mRNAs at low temperature, which are likely incompatible with translation initiation and elongation, may play a major role in translational silencing. Furthermore, it has been reported that the massive amount of RNA-binding cold shock proteins, which accumulates in stressed cells, overcrowds the mRNAs. In turn, this causes a generalized inhibition of bulk protein synthesis and mRNA degradation by RNaseR, whereas the translation of only a select group of cold-shock transcripts is favored by the RNA-chaperone activity of the Csp proteins (Ermolenko and Makhatadze, 2002; Hofweber et al., 2005; Zhang et al., 2018).

A more selective role in discriminating against translation of non-cold shock mRNAs such as *cspD* mRNA is played by IF3. It has been shown in this case that 30SIC formed with this mRNA at cold-shock temperature is endowed with a non-canonical structure and is rejected by IF3 before becoming productive (Gualerzi et al., 2011).

It has been postulated that cold-shock-induced protein PY could be responsible for the repression of bulk translation at the onset of cold adaptation (Agafonov et al., 2001; Vila-Sanjurjo et al., 2004). In accordance with this hypothesis, analysis of the phenotypes of *E. coli* PY deletion mutants (Di Pietro et al., 2013) confirmed that PY diminishes bulk translation, reduces translation of some mRNAs, non-cold-shock mRNAs in particular, and influences the timing of cold-shock induction of some proteins. In addition, the data indicated that, although PY cannot destabilize an fMet-tRNA correctly positioned in the 70SIC, its main target at low temperature is translation initiation rather than elongation as previously suggested. However, the extent of translation inhibition by PY was rather modest and recovery from cold stress and inhibition of bulk protein synthesis following cold stress occurred also in the absence of PY. Given the vital importance of blocking bulk translation upon cold shock, it is likely that in the absence of PY the cell may resort to some back up mechanism to reduce translational activity, possibly by overcrowding the mRNAs with large amounts of cold shock-induced RNA-binding proteins. In this connection, PY was shown to reduce the number of ribosomal subunits able to participate in 70SIC formation by sequestering 30S and 50S subunits and stimulating their association to form idle 70S monomers (Figure 7).

Among the proteins whose expression is increased after cold stress are well known DNA binding proteins such as RecA, GyrA/GyrB (Jones et al., 1992), H-NS (La Teana et al., 1991), and HUβ (Giangrossi et al., 2002). It is known that temperature changes affect the degree of DNA superhelicity and the compaction of the nucleoid and it takes several hours after cold stress before the DNA superhelicity reaches an equilibrium (Goldstein and Drlica, 1984), likely corresponding to the time required for the cold-shock-induced gyrase synthesis. Thus, the gyrase (GyrA/GyrB) and HU, which introduce negative supercoiling (Drlica, 1992; Dorman, 1996; Tse-Dinh et al., 1997), and H-NS, which constrains the DNA superhelicity (Dorman, 1996; Atlung and Ingmer, 1997) could play an important role in the structural rearrangements of the nucleoid which occur upon temperature downshift.

However, it is possible to envisage an additional, perhaps more elusive role in mRNA translation for the cold-induced nucleoid-associated proteins HU and H-NS. Indeed, both HU and H-NS have been shown to affect protein synthesis in particular cases.

**FIGURE 8:**
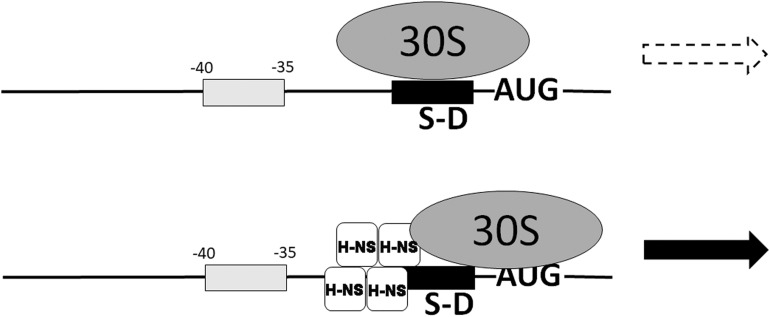
Nucleoid-associated transcriptional inhibitor H-NS can promote translation of some mRNAs. The translation of some mRNAs characterized by a weak SD sequence and an A,T-rich box between –35 and –40 is stimulated by nucleoid-associated protein H-NS which places the 30S ribosomal subunit in a new position, promotes 30SIC formation and stimulates translation (Park et al., 2010). H-NS is bound to the mRNA near the 30S ribosomal subunit and represented as a tetramer which is the active form of this protein, according to our studies (Spurio et al., 1997; Stella et al., 2005). Modified from Park et al. (2010).

Translation of *malT* mRNA is stimulated by H-NS which facilitates the assembly of a 30S initiation complex (30SIC) on this mRNA whose 5′UTR possesses some particular properties consisting in a suboptimal ribosome-binding sequence and in the presence of an AU-rich region at −35 to −40 (Figure 8). In this connection it should be remarked that AU rich enhancers stimulate translation in co-operation with the SD sequences and that shorter SD sequences are preferred at lower temperatures (Vimberg et al., 2007). It seems noteworthy that H-NS stimulates also the translation of other mRNAs whose 5′UTRs have similar characteristics (Park et al., 2010). However, among the large number of genes whose expression is stimulated by cold shock (Zhang et al., 2018) the 5′UTR of only four ORFS (*ymgA*, *yehU*, *yrhB*, and *ylbH*) have characteristics similar to typical H-NS targets.

Like H-NS, also HU was shown to influence the level of translation, at least in one particular case. Upon binding with high specificity to the *rpoS* mRNA, HU was found to stimulate the synthesis of the stress σ^*S*^ factor of RNA polymerase (Balandina et al., 2001). It seems interesting in this connection that HU and H-NS have antagonistic effects on the expression of σ^*S*^. In fact, HU binds DsrA, a small non-coding RNA that regulates transcription by repressing H-NS and stimulates translation by increasing expression of *rpoS*.

These results are in accordance with earlier findings that during the acclimation phase of cold shock the protein expression pattern of the organism drastically changes.

## Conclusion and Perspectives

The translational apparatus plays an essential role in the production of vital cell constituents like proteins. In addition to this primary function, the translational apparatus plays an active role in reprogramming gene expression and proteome composition, as illustrated by the mechanisms by which bacteria cope with the three stress situations outlined above. Although considerable amount of work has been devoted to clarify the events occurring in the cells following zinc and nutrient shortage and cold stress, several aspects of cellular responses to these stresses deserve further investigation. The signals which promote the replacement of the Zn-containing ribosomal proteins by their paralogs and the mechanisms by which this replacement takes place and the subsequent steps leading to an increase of free zinc availability in the cytoplasm should be clarified. The mechanism by which translation of a select group of mRNAs is favored by ppGpp remains unclear. In particular, it would be important to understand what is responsible for the modulation of IF2 affinity for GTP and ppGpp. In light of the recent findings that several small non-coding RNAs (sRNAs) may affect translation in *cis* or in *trans*, thereby providing effective responses to different types of stress (e.g., metabolite, nutrient, oxidative, envelope, iron, acidic, anaerobic, and heat stresses) (Citartan et al., 2016; Jagodnik et al., 2016), it would be important to investigate if sRNAs may also play a role in cold shocked cells. Concerning the cold shock-induced modifications of the translational apparatus, it should be determined if cold stress alters the RNA post-transcriptional modification patterns and if the post-translational modifications of the ribosomal proteins observed in cold shocked cells (Piersimoni, 2010) have any functional implication. Also the role, if any, played by cold-shock RNA binding proteins other than CspA in inhibiting bulk protein synthesis and favoring translation of cold-shock transcripts should be investigated. Finally, a thorough characterization of cold-adapted cells which grow and divide at suboptimal temperatures is still lacking.

## Author Contributions

HC-G and CG contributed to writing the MS. Both authors contributed to the article and approved the submitted version.

## Conflict of Interest

The authors declare that the research was conducted in the absence of any commercial or financial relationships that could be construed as a potential conflict of interest.
